# Interleukin-6 producing pheochromocytoma/paraganglioma: case series from a tertiary referral centre for pheochromocytomas and paragangliomas

**DOI:** 10.1007/s40618-021-01532-5

**Published:** 2021-03-14

**Authors:** A. C. Meijs, M. A. Schroijen, M. Snel, E. P. M. Corssmit

**Affiliations:** 1 Department of Medicine, Division of Endocrinology, Leiden University Medical Centre, Albinusdreef 2, 2333 ZA Leiden, The Netherlands; 2 Centre for Endocrine Tumours Leiden (CETL), Leiden University Medical Centre, Albinusdreef 2, 2333 ZA Leiden, The Netherlands

**Keywords:** Pheochromocytoma, Paraganglioma, Interleukin-6, Catecholamines

## Abstract

**Introduction:**

In addition to catecholamines, pheochromocytomas and paragangliomas (PPGL) may secrete interleukin-6 (IL-6). IL-6 contributes to the development of unusual symptoms, which may hinder the diagnosis.

**Patients and methods:**

We report the clinical course and subsequent treatment of IL-6 producing PPGL in three patients from a single tertiary referral centre for PPGL patients in the Netherlands.

**Conclusion:**

PPGL combined with persistent elevated inflammatory markers, either in the presence or absence of pyrexia, raised suspicion of IL-6 overproduction in these three patients. Although surgical resection of the tumour is the only curative treatment option, our case series adds to the accumulating evidence that alpha-blockers might be effective in these patients.

## Introduction

Pheochromocytomas and paragangliomas (PPGL) are rare neuroendocrine tumours arising from chromaffin cells of the adrenal medulla and the extra-adrenal neural crest progenitors, respectively, which may secrete catecholamines [1]. The estimated annual incidence of PPGL is 0.8 per 100.000 person-years [2], although PPGL are more prevalent among patients with hypertension (0.1%) [3]. PPGL may cause a wide variation of symptoms due to excessive catecholamine release, such as episodic headache, diaphoresis, tachycardia, and sustained or paroxysmal hypertension [1, 4, 5]. Other bioactive neuropeptides or hormones secreted by some PPGL may contribute to developing unusual symptoms, thereby hindering the diagnosis. One of these peptides is interleukin-6 (IL-6), a pleiotropic cytokine with pivotal roles in immune and inflammatory responses [6]. IL-6 stimulates differentiation of B cells, activation of T cells and regulates synthesis of acute phase proteins such as C-reactive protein [7, 8]. In addition, overproduction of IL-6 leads to an inflammatory syndrome in PPGL [9].

To date, 42 cases of IL-6 producing PPGL have been described. In this case series, we describe three patients with IL-6 producing PPGL aiming to identify the clinical, biochemical, radiological and genetic characteristics associated with this rare presentation of PPGL and evaluate subsequent treatment options.

## Patients and methods

In this retrospective single-centre case series, medical records were assessed of patients with IL-6 producing PPGL from the Leiden University Medical Centre (LUMC), a tertiary referral centre for PPGL in the Netherlands. Clinical characteristics including sex, age, clinical presentation, laboratory findings, radiologic imaging, clinical course and treatment of the IL-6 producing PPGL were collected. Patients were screened for germline mutations in succinate dehydrogenase (SDH) A, -B, -C, -D, -AF2, Transmembrane protein 127 (TMEM127), MYC-associated protein X (MAX), Von Hippel–Lindau (VHL) and REarranged during Transfection (RET) by sequencing analysis (Ion AmpliSeq PGL Community Panel kit), if needed supplemented with Sanger Sequencing. In addition, Multiplex Ligation-dependent Probe Amplification (MPLA) was performed for detection of deletions and duplications in SDHB, -C, -D, -AF2 (MRC-Holland kit P226) and VHL (MRC-Holland kit P016) [10]. Written informed consent regarding publishing the data and images was obtained from each participant.

## Results

Within a cohort of 422 patients who underwent surgery for a sporadic or syndromic PPGL between 2002 and 2020 in the LUMC, three patients with IL-6 producing PPGL were identified from patient records. The patients are described in more detail below (Table 1).

**Table 1:** Patient characteristics

	Patient 1	Patient 2	Patient 3
Age at presentation	27	26	60
Gender	Male	Female	Male
Genetic testing	SDHD^a^ mutation (c.274G > T p.(Asp92Tyr))	SDHD^a^ mutation (c.169_169 + 9delTGTATGTTCT, splice donor defect in exon 2), in addition SDHA^b^ mutation (c.1771G > A p.(Ala591Thr) in exon 13), variant of uncertain significance (VUS)	Negative
Family history PPGL	Positive	Negative	Negative
Number of PPGL	5	2	1
Location PPGL	2 CBT^c^, 3 para-aortic PGL	CBT^c^ and VBT^d^	Right adrenal
Size of PPGL, cm	CBT^c^: 2.5 × 5.6 × 8.5 (right) and 1.8 × 2.6 × 4.1 (left) Para-aortic PGL: 1.0, 2.0 and 2.0	CBT^c^: 3.6 × 2.9 × 2.9 VBT^d^: 5.1 × 3.3 × 3.7	13.0 × 9.5 × 9.1
Metastases	Lesions suspicious for pulmonary and liver metastases	No	No

^a^*SDHD* succinate dehydrogenase complex subunit D

^b^*SDHA* succinate dehydrogenase complex flavoprotein subunit A

^c^*CBT* carotid body tumour

^d^*VBT* vagal body tumour

### Patient 1

In 2012, a 21-year-old man was analysed at the outpatient clinic of the department of endocrinology after referral by the department of clinical genetics. His father and 22-year-old brother tested positive for succinate dehydrogenase complex subunit D (SDHD) mutation and his brother had one abdominal and two thoracic paragangliomas. Genetic analysis in the patient showed a SDHD mutation [c.274G > T p.(Asp92Tyr)]. His medical history was unremarkable and he was not on any medication. In addition, he did not have any PPGL-related symptoms. Physical examination showed no abnormalities besides a slightly elevated blood pressure of 146/75 mmHg. 24 h urinary excretion of normetanephrine was increased (670 μmol/mol creatinine, reference range 64–260). MRI scan disclosed two carotid body tumours of 4.2 × 5.0 × 6.8 and 1.6 × 2.1 × 3.6 cm and a para-aortic mass. 123I-metaiodobenzylguanidine (MIBG) scan showed accumulation of the isotope in the three masses, not elsewhere. Laparoscopic surgical resection of the intra-abdominal mass was performed, which histopathologically proved to consist of two separate paragangliomas of 1 and 2 cm. Postoperatively, urinary normetanephrine excretion was normal.

Four years later, in 2016, the patient presented with an elevated erythrocyte sedimentation rate (ESR) (72 mm) and C-reactive protein (CRP) (56.1 mg/L), thrombocytosis (501 × 10^9/L) and anaemia (haemoglobin 7.4 mmol/L). MCV was 78 fL, indicating slightly microcytic anaemia (Table 2). He did not have any symptoms of clinical significance and the body temperature was normal. 24 h urinary excretion of metanephrines was normal; additional gastroscopy, bone marrow biopsy and tests for infectious and auto-immune diseases revealed no abnormalities. However, an 18FDG-PET scan exhibited two intra-abdominal masses: one located at the right adrenal gland and one at the position of the previously resected para-aortic paraganglioma. 123I-MIBG scan and Indium-111-octreotide scintigraphy did not show accumulation of MIBG or octreotide in the abdominal masses. The abdominal masses were resected via laparoscopic surgery. Histopathological examination confirmed that the para-aortic mass was a new or relapsed paraganglioma; the right adrenal gland showed hyperplasia but no clear pheochromocytoma.

**Table 2:** Laboratory results

	Patient 1	Patient 2	Patient 3		Reference range
			Before^a^	After^a^	
Interleukin-6, pg/mL	23.0	12.7	73.6, after start of doxazosin 9.55	17.7	Patient 1: 0.43–8.87 Patient 2 and 3: 0.50–3.92
24 h urinary metanephrines					
Normetanephrine, μmol/mol creatinine	161	133	2896	147	25–280
Metanephrine, μmol/mol creatinine	44	65	3952	47	20–110
3-M-tyramine, μmol/mol creatinine	109	587	579	94	20–200
Erythrocyte sedimentation rate, mm	72	60–90	94	2	0–15
C-reactive protein, mg/L	56.1	48.0	186.2	1.0	< 5.0
Thrombocytes, 10^9/L	501	458	668	328	150–400
Leucocytes, 10^9/L	9.38	8.60	17.79	9.03	4.00–10.00
Haemoglobin, mmol/L	7.4	7.3	7.7	8.9	Patient 1 and 3: 8.5–11.0; Patient 2: 7.5–10.0
Mean corpuscular volume, fL	78	76	90	86	80–100

^a^Laboratory values before and after adrenalectomy/stopping doxazosin

Postoperatively, the initial abnormal laboratory findings did not return to normal. In the workup of inflammation in a patient with known paragangliomas, IL-6 was measured and found to be elevated (23.0 pg/mL, reference range 0.43–8.87 pg/mL). A 68 Ga-DOTATATE PET-CT revealed a 0.5 cm pulmonary lesion, negative on 18FDG-PET scan and 131I-MIBG scan, suspicious for metastasis; 18FDG PET-CT scan showed a new 0.5 cm hepatic mass, negative on 68 Ga-DOTATATE PET-CT scan and 131I-MIBG scan, also suspicious for metastasis. We concluded that the increased inflammatory parameters might result from IL-6 production by either (one of the) head neck paragangliomas or the newly diagnosed lesions suspicious for metastases. During follow-up, a wait-and-scan strategy according to RECIST 1.1 criteria was chosen. It showed that all lesions were stable, and, therefore, no additional treatment was initiated. The patient did not develop fever or other signs of inflammation. Up till present, IL-6 and other inflammatory parameters have remained elevated.

### Patient 2

**Fig. 1:**
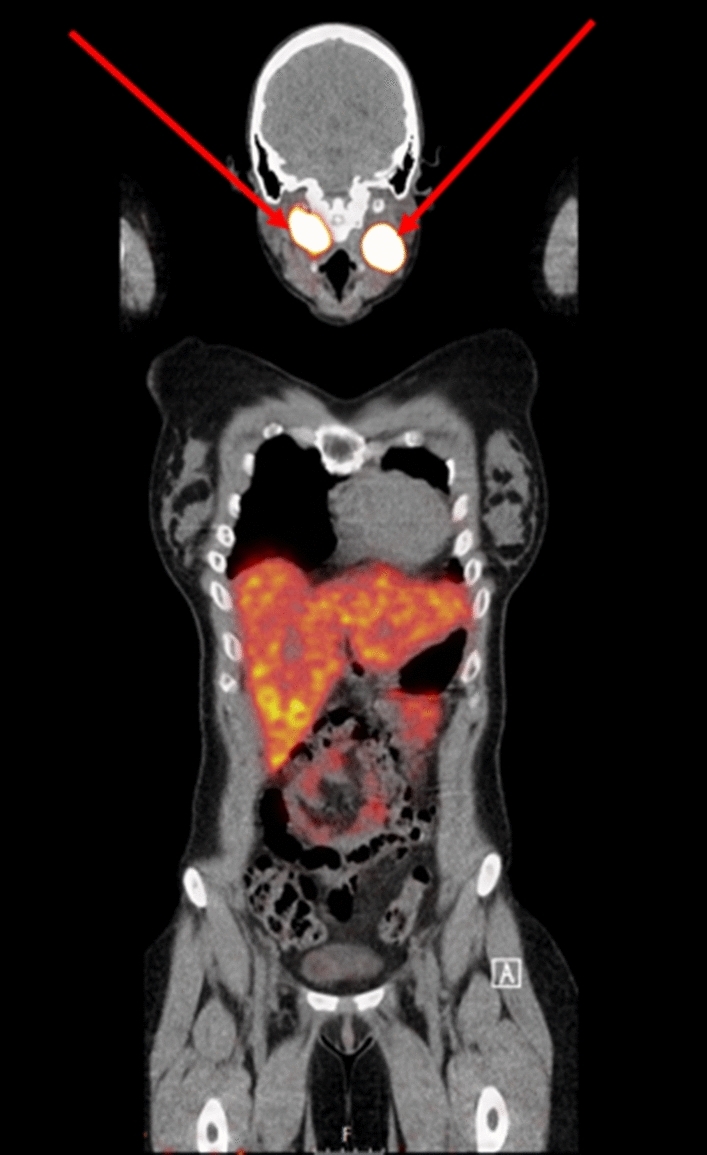
68 Ga-DOTATATE PET-CT scan demonstrating increased uptake in a right-sided vagal body tumour and a left-sided carotid body tumour in patient 2

In 2019, a 26-year-old female patient presented at the department of internal medicine with persistent fatigue, paroxysmal palpitations, night sweats and alternating hot and cold experiences, but normal body temperature on repeated measurements. Her medical history included a central muscular ventricular septal defect detected at the age of three, heterozygous thalassemia, and a recent giardiasis infection for which metronidazole was administered. Laboratory findings showed an elevated ESR of 60 mm and CRP of 48 mg/L and thrombocytosis (458 × 10^9/L). Haemoglobin was 7.3 mmol/L and MCV 76 fL (Table 2). An 18FDG PET-CT scan showed increased uptake at two locations in the oropharynx, which was compatible on MRI with a right-sided vagal body tumour of 5.1 × 3.3 × 3.7 cm and a left-sided carotid body tumour of 3.6 × 2.9 × 2.9 cm. No other PPGL were detected on abdominal and thoracic MRI. An additional 68 Ga-DOTATATE PET-CT scan showed increased uptake in the two masses, but not elsewhere (Fig. 1). 24 h urine collection demonstrated elevated excretion of 3-M-tyramine (587 μmol/mol creatinine, reference range 20–200) and normal excretion of normetanephrine and metanephrine.

Extensive additional investigations were performed because of the persistent elevation of ESR and CRP, which ruled out chronic infections, including TBC and endocarditis, and auto-immune diseases including SLE and sarcoidosis. Additional cardiologic evaluation revealed a hemodynamical non-significant ventricular septal defect on echocardiography, but no other abnormalities. Thereafter, the combination of unexplained inflammation and the presence of paragangliomas raised the suspicion of an IL-6 producing paraganglioma. Measurement of plasma IL-6 concentration showed a marked elevated level of 12.7 pg/mL (reference range 0.50–3.92 pg/mL). Ibuprofen and pantoprazole were advised, but not used by the patient. Surgical resection of the carotid body tumour is under evaluation. As resection of the vagal body tumour will cause vocal cord arrest, this tumour will be followed on MRI scans. Although family history was negative, genetic analysis showed a SDHD mutation (c.169_169 + 9delTGTATGTTCT, splice donor defect in exon 2), confirming the diagnosis of hereditary paraganglioma/pheochromocytoma syndrome type 1 (PGL1). In addition, a SDHA mutation was found [c.1771G > A p.(Ala591Thr) in exon 13], a variant of uncertain significance (VUS).

### Patient 3

In 2019, a 60-year-old man presented at the emergency department with a fever (body temperature rising to 39 °C) for more than 2 weeks. His medical history included basal cell carcinoma and eczema. He did not have localizing signs or symptoms except for a mild headache and muscle soreness in his legs. Physical examination revealed a grade 2 systolic murmur, no other abnormalities.

**Fig. 2:**
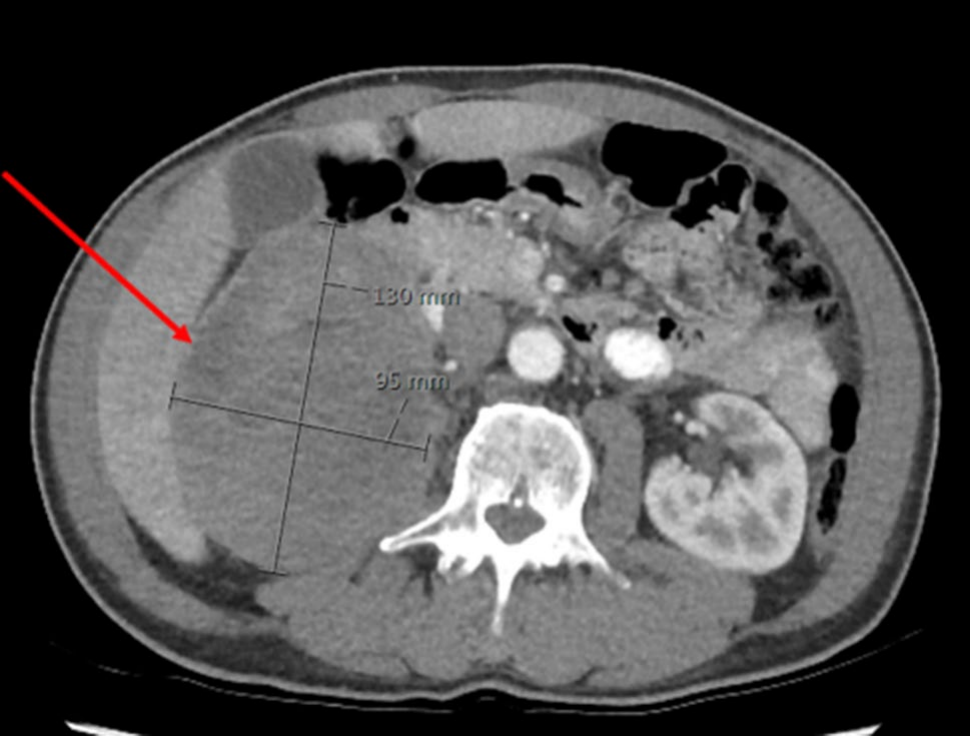
Abdominal CT scan demonstrating a mass of 13 cm originating from the right adrenal gland in patient 3

**Fig. 3:**
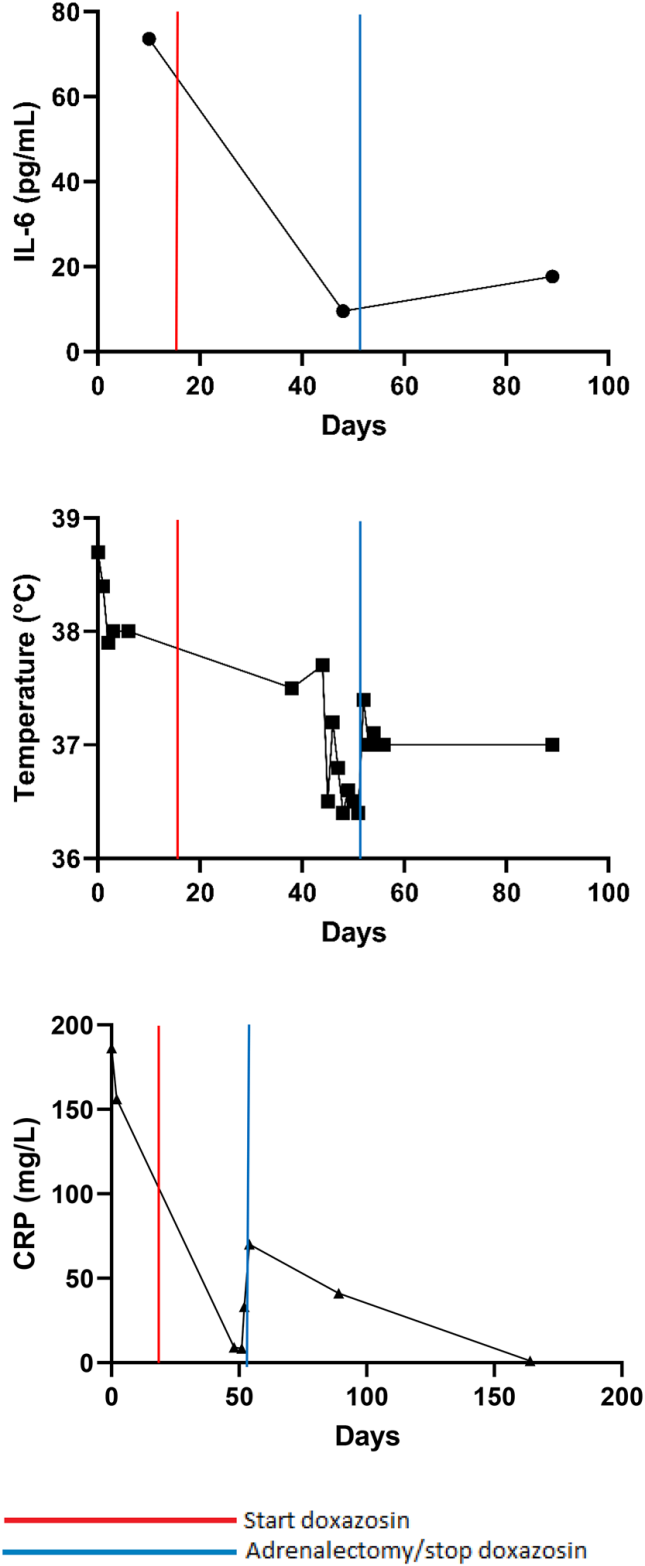
Course of interleukin-6 (IL-6, reference range 0.50–3.92 pg/mL), body temperature and C-reactive protein (CRP, reference range < 5.0 mg/L) in patient 3

Laboratory findings showed elevated inflammatory markers, including an ESR of 94 mm and a CRP of 186.2 mg/L. Furthermore, normocytic anaemia was detected (Hb 7.7 mmol/L, MCV 90 fL), as were thrombocytosis (668 × 10^9/L) and leucocytosis (17.79 × 10^9/L) (Table 2). Extensive additional testing was initiated, including bacterial cultures, hepatitis viral markers, quantiferon, M-protein, serologic tests for HIV, borrelia and syphilis, and an immunoglobulin panel, which were all negative. Chest and sinus radiography, echocardiography and ECG were normal. However, 18FDG PET-CT scan showed a mass of either the right kidney or adrenal gland. Abdominal CT confirmed that the mass of 13.0 × 9.5 × 9.1 cm most likely originated from the right adrenal gland and was suspect for a pheochromocytoma or an adrenal cortex carcinoma (Fig. 2). No metastases were seen on radiological imaging. 24 h urine collection revealed increased excretion rate of metanephrine (3952 μmol/mol creatinine, reference range 20–110), normetanephrine (2896 μmol/mol creatinine, reference range 25–280) and 3-M-tyramine (579 μmol/mol creatinine, reference range 20–200), consistent with the diagnosis of a functional pheochromocytoma. The combination of pheochromocytoma with persistent fever and elevated inflammatory markers raised the suspicion of IL-6 production. IL-6 level appeared to be strongly elevated (73.6 pg/mL, reference range 0.50–3.92 pg/mL). Open adrenalectomy of the right adrenal gland was scheduled. After the start of preoperative alpha blockade using doxazosin, general malaise attenuated, body temperature decreased to 37.5 °C and IL-6 level decreased but was still elevated (9.55 pg/mL, reference range 0.50–3.92 pg/mL). Per- and post-operative course were uncomplicated. Histopathology confirmed the diagnosis of a pheochromocytoma. Pheochromocytoma of the Adrenal Gland Scaled Score (PASS) was 3 suggesting a benign lesion. Postoperatively, doxazosin was stopped. Body temperature, ESR, CRP, thrombocytes and haemoglobin level gradually returned to normal. In addition, follow-up revealed normalization of urinary excretion of metanephrines and an IL-6 level of 17.7 pg/mL (Fig. 3). The patient was referred to the department of clinical genetics to investigate possible genetic causes of this large pheochromocytoma. However, genetic testing for known germline mutations was negative.

## Discussion

In this case series, three patients with IL-6 producing PPGL are described, including a patient with multiple paragangliomas with lesions suspicious for metastases, a patient with head neck paragangliomas and a patient with a large pheochromocytoma. To date, 39 cases with IL-6 producing pheochromocytoma and three cases with IL-6 producing paraganglioma have been reported [11–13]. However, the actual incidence of IL-6 producing PPGL might be higher as it may frequently be overlooked due to unawareness of the diagnosis, or clinical manifestations may be masked by the excessive secretion of catecholamines [13].

To our knowledge, we report the first patients with increased IL-6 production in the presence of SDHD mutation related paragangliomas and lesions suspicious for metastases. Two of the previously reported patients with IL-6 producing PPGL had metastatic disease, which originated from paraganglioma [11, 13]. However, previously reported patients with IL-6 producing PPGL did either have no mutation or were not screened for gene mutations. Metastatic disease is quite rare in patients with a SDHD mutation: a systematic review and meta-analysis reported a 4% pooled risk of malignant PPGL in SDHD mutation carriers in the prevalence studies [14]. An association between a loss of function SDHD germline mutation and IL-6 has not been described before. On the contrary, inhibition of succinate dehydrogenase has been shown to be anti-inflammatory in vivo [15].

The exact origin of excessive IL-6 secretion in the patients with PPGL needs to be elucidated. It has been suggested that IL-6 can be secreted directly by the tumour [16]. Cheng et al*.* reported that immunohistochemically measured expression of IL-6 protein in pheochromocytoma tissue was significantly higher in patients with pyrexia and IL-6 excess than in patients with normal body temperature and low IL-6 levels [6]. Similarly, other studies reported IL-6 expression on resected PPGL of patients with increased IL-6 levels and increased inflammatory markers [7, 11, 17–21]. It has also been suggested that IL-6 can be secreted by the tumour as a consequence of high circulating norepinephrine levels [16]. However, Cheng et al*.* reported that several of their pheochromocytoma patients with IL-6 overproduction did not have norepinephrine excess [6]. Previously reported IL-6 producing paragangliomas did also not show catecholamine excess, in line with our paraganglioma patients who had, like most head neck paragangliomas and SDHD mutation carriers [22], normal urinary normetanephrine excretion. Therefore, it is more presumable that IL-6 is predominantly synthesized and secreted by PPGL neoplastic cells than as a consequence of excessive norepinephrine levels exclusively.

Previously described patients with IL-6 producing pheochromocytomas, as well as paragangliomas, mostly presented with pyrexia that was resistant to any treatment, but resolved after surgical resection of the tumour [13]. In our case series, only patient 3 presented with pyrexia. One patient did have alternating hot and cold experiences, but body temperature was not elevated on repeated measurements. Nevertheless, IL-6 is known to serve as an endogenous pyrogen, which may result in the very rare presentation of PPGL with fever of unknown origin (FUO) [6]. FUO has been defined as fever (≥ 38.3 °C) for more than 3 weeks that remains undiagnosed after a hospital workup [23]. Besides PPGL, more than 200 diseases are linked to FUO [9].

Pyrexia in these patients is caused by IL-6 that crosses the blood–brain barrier and initiates synthesis of prostaglandin E_2_ (PGE_2_) in the hypothalamus, which results in changing the setpoint of body temperature [24]. It has been reported that injection of recombinant IL-6 in rats and rabbits caused pyrexia [25, 26], and that IL-6 was significantly higher in pheochromocytoma patients with high body temperature than in pheochromocytoma patients with normal body temperature [6].

Laboratory abnormalities in our patients, including elevated plasma concentrations of inflammatory markers, anaemia, thrombocytosis and leucocytosis, were in accordance with previously described cases and can be attributed to elevated IL-6 levels [20]. CRP and ESR were markedly elevated in our patients in the presence of increased levels of IL-6, a potent inducer of inflammatory markers. Furthermore, it has been demonstrated that cytokines such as IL-6 inhibit erythroid progenitor proliferation and impair iron supply to developing erythroid cells, resulting in anaemia associated with chronic inflammation [18]. Besides anaemia, other hematologic laboratory abnormalities including leucocytosis and thrombocytosis may develop due to overproduction of IL-6. This is consistent with the notion that IL-6 has been implicated in polyclonal B cell activation, differentiation from B cells to plasma cells, and stimulation of developing megakaryocytes [20]. Other studies reported the normalization of laboratory values, including IL-6 levels, after resection of the tumour. This supports the hypothesis that these laboratory abnormalities are a result of IL-6 overproduction [13], which is in line with our patient 3, whose laboratory findings and clinical condition improved after adrenalectomy.

Surgical resection of the tumour is the only curative treatment for (IL-6 producing) PPGL. However, it has been suggested that pharmacological treatment options including non-steroidal anti-inflammatory drugs (NSAIDs) and alpha-blockers can reduce clinical manifestations of IL-6 overproduction.

Two case reports showed that naproxen was effective in treating fever in patients with IL-6 producing pheochromocytoma [20, 27]. When naproxen was discontinued in one of these patients, the febrile state immediately returned to the pre-treatment level, which suggests that naproxen impacts on IL-6 activity [27]. Similarly, Shimizu et al*.* reported that IL-6 levels and other laboratory markers normalized concurrent with lowering body temperature after administering naproxen in a patient with IL-6 producing pheochromocytoma. Additionally, they demonstrated that naproxen reduced IL-6 secretion in vitro [8]. Correspondingly, Tokuda et al*.* reported that body temperature and CRP significantly decreased after administration of loxoprofen in a patient with IL-6 producing pheochromocytoma [7]. Clinical effectiveness of NSAIDs in IL-6 producing paraganglioma may be explained by experiments using a human astrocytoma cell line that revealed that naproxen affects post-translational modification of IL-6 protein or secretory processes [28]. Furthermore, it has been reported that the reduction of PGE_2_, which is induced by cyclo-oxygenase (COX) 2 inhibitors such as naproxen, affects IL-6 production and secretion in rat adjuvant arthritis [29]. However, one case report showed that the administration of NSAIDs was ineffective, whereas a corticosteroid agent was effective in lowering body temperature and attenuating general malaise [18].

Besides NSAIDs, alpha-blockers have also been suggested as an effective pharmacological treatment option in IL-6 producing PPGL. Two case reports showed that after the administration of an alpha-blocker fever resolved and IL-6 levels decreased [16, 30]. Investigation of the anti-inflammatory effects of doxazosin, a long-acting α1-adrenergic receptor antagonist, revealed that doxazosin inhibited tumour necrosis factor-α (TNF-α) and monocyte chemoattractant protein-1 (MCP-1) production in mice [31] and might, therefore, be effective in patients with IL-6 producing PPGL. Hence, the anti-inflammatory effects of doxazosin might have contributed to the decrease in body temperature, improvement of the clinical condition and reduction of IL-6 level before surgery in our patient 3.

In conclusion, we report three patients with increased IL-6 levels and inflammatory markers in the presence of PPGL. Combination of persistent elevated inflammatory markers, either in the presence or absence of pyrexia, and the presence of PPGL, raised suspicion of IL-6 overproduction in our patients. Although surgical resection of the tumour is the only curative treatment option, our case series adds to the accumulating evidence that alpha-blockers might be effective and an alternative for treatment with NSAIDs in these patients.
